# Synergistic Effects of Ethanol and Isopentenyl Pyrophosphate on Expansion of γδ T Cells in Synovial Fluid from Patients with Arthritis

**DOI:** 10.1371/journal.pone.0103683

**Published:** 2014-08-04

**Authors:** Agneta J. Laurent, Niels Bindslev, Björn Johansson, Louise Berg

**Affiliations:** 1 Rheumatology Unit, Department of Medicine, Karolinska Institutet, Stockholm, Sweden; 2 Department of Biomedical Sciences, Faculty of Health and Medical Sciences, University of Copenhagen, Copenhagen, Denmark; 3 Neurogenetics Unit, Department of Molecular Medicine and Surgery, Karolinska Institutet, Stockholm, Sweden; Ohio State University Medical Center, United States of America

## Abstract

Low to moderate ethanol consumption has been associated with protective effects in autoimmune diseases such as rheumatoid arthritis, RA. An expansion of γδ T cells induced by isopentenyl pyrophosphate, IPP, likewise seems to have a protective role in arthritis. The aim of this project was to test the hypothesis that low doses of ethanol can enhance IPP-induced expansion of synovial fluid γδ T cells from patients with arthritis and may thereby potentially account for the beneficial effects of ethanol on symptoms of the arthritic process. Thus, mononuclear cells from synovial fluid (SF) from 15 patients with arthritis and from peripheral blood (PB) from 15 healthy donors were stimulated with low concentrations of ethanol and IPP for 7 days *in vitro*. IPP in combination with ethanol 0.015%, 2.5 mM, equivalent to the decrease per hour in blood ethanol concentration due to metabolism, gave a significantly higher fractional expansion of SF γδ T cells compared with IPP alone after 7 days (ratio 10.1+/−4.0, p<0.0008, n = 12) in patients with arthritis. Similar results were obtained for PB γδ T cells from healthy controls (ratio 2.0+/−0.4, p<0.011, n = 15). The augmented expansion of γδ T cells in SF is explained by a higher proliferation (p = 0.0034, n = 11) and an increased survival (p<0.005, n = 11) in SF cultures stimulated with IPP plus ethanol compared to IPP alone. The synergistic effects of IPP and ethanol indicate a possible allosteric effect of ethanol. Similar effects could be seen when stimulating PB with ethanol in presence of risedronate, which has the ability to increase endogenous levels of IPP. We conclude that expansion of γδ T cells by combinatorial drug effects, possibly in fixed-dose combination, FDC, of ethanol in the presence of IPP might give a protective role in diseases such as arthritis.

## Introduction

Rheumatoid arthritis, RA, is a systemic, inflammatory, autoimmune disorder [1] affecting about 0.5–1% of the adult population worldwide [2]. It is characterized by inflammation in joints causing both bone and cartilage destruction [1]. The destruction of bone and cartilage is dependent on different mechanisms in which autoantibodies, osteoclasts, activated T cells and fibroblast-like synoviocytes are involved [1]. Fibroblast-like synoviocytes (FLS) residing in the inner synovial lining of two cell layers, which together form the healthy synovial membrane, produce synovial fluid to the inner cavity of the joint [2]. RA pathogenesis is characterized by inflammatory cell infiltration, supported by proliferation of FLS and angiogenesis. Synovial hyperplasia eventually develops a so called pannus tissue, which invades and destroys the cartilage and the underlying bone [2].

Low to moderate alcohol consumption has been shown to have protective effects in several chronic inflammatory diseases such as rheumatoid arthritis and cardiovascular diseases [3]. Epidemiological studies have analyzed the risk factor of alcohol consumption in developing rheumatoid arthritis and showed a preventive effect of alcohol in rheumatoid arthritis [4]–[13]. In a mouse model of arthritis, ethanol has been shown to lower the chemotactic activity of leukocytes and down-regulate NF-κB activity [14]. The molecular mechanisms behind the protective effects of low to moderate alcohol consumption in humans remain to be elucidated.

T cells bearing the γδ T cell receptor normally constitute 2–5% of peripheral blood T cells [15], are evolutionary conserved, and have specific tissue distribution and functions [16]. There are two major γδ T cell subsets, Vδ1 and Vδ2, of which the Vδ2 T cells are the most common. Vδ2 T cells are predominantly found in peripheral blood, and Vδ1 T cells are most abundant in gut mucosa [16]. In contrast to αβ T cells the activation of γδ T cells is not dependent on antigen presentation by major histocompatibility complex (MHC) class I or II molecules. Instead, Vδ1 T cells respond to stress-induced cell-surface major histocompatibility class-I related proteins MICA and MICB [16], while Vδ2 T cells proliferate in response to prenyl pyrophosphates such as isopentenyl pyrophosphate (IPP), an intermediate in the mevalonate pathway, with an EC_50_ of 1 µM [15]. Further, Vδ2 T cells are stimulated by (*E*)-4-hydroxy-3-methyl-but-2-enyl pyrophosphate (HMBPP), produced by bacteria and parasitic protozoa, with an even higher potency of EC_50_ = 32 pM [15]. In an indirect mechanism, Vδ2 T cells are also stimulated by bisphosphonates and alkylamines, in part by increasing intracellular levels of IPP [15], [17].

It has been suggested that rheumatoid arthritis is associated with alterations in the different subsets of γδ T cells [18]. In RA, γδ T cells constitute a smaller fraction of total T cells in blood and synovial fluid (SF) compared to controls. In addition, the ratio of Vδ2/Vδ1 T cells in blood and synovial fluid is lower in RA patients compared to controls [19]. Moreover a greater proportion of IPP responsive γδ T cells have been found in patients who recovered from disease [20].

Fibroblast-like synoviocytes (FLS) in the synovium of patients with RA show abnormal behavior including increased proliferation and production of proinflammatory mediators and enzymes [21]. Thus, it has recently been shown that IPP stimulated SF γδ T cells can induce apoptosis of FLS *in vitro* [22], which could argue for a protective role of γδ T cells in the inflamed joint. Furthermore, an increased ratio of SF γδ T cells in proportion to FLS increased apoptosis of FLS [23].

Evidently, ethanol potentiates proliferation of mitogen-activated T lymphocytes in blood from humans [24]. However, studying effects of ethanol on proliferation of T lymphocytes from *synovial fluid* seems more relevant for future alcohol related treatment of arthritic symptoms. We therefore asked whether ethanol may have an enhancing effect on IPP stimulated synovial fluid γδ T cells. We also asked if there is an additive or synergistic mechanism behind possible ethanol-enhancing effects on expansion with IPP. Our results show that a low dose of ethanol can indeed enhance the expansion of IPP stimulated SF γδ T cells *in vitro*, and that this ethanol enhancing effect is synergistic at certain IPP-ethanol concentration combinations.

## Materials and Methods

### Patients

Of eighteen patients included in this study, seventeen were attending the Rheumatology Unit at Karolinska University Hospital in Solna and one at the University Hospital in Uppsala. Clinical characteristics and medication of patients are shown in table 1. 15 healthy volunteers, 9 women and 6 men, median age 46 (27–79) years were included. The study was approved by The Regional Ethical Review Board in Stockholm (Regionala Etikprövningsnämnden i Stockholm), number 2010/351-31/2. At the time of sampling, patients were informed of the study and of the possibility of donating samples to research. Oral consent was obtained and documented by the physician in the patients' electronic records, as is the normal procedure in Sweden. The oral consent was in accordance with Swedish law and practice and approved by The Regional Ethical Review Board in Stockholm.

**Table 1:** Characteristics of the 18 patients participating in the study.

Patient	Diagnosis	Sex	Age	Treatment
1	Psoriatic arthritis	F	53	Prednisone
2	Psoriatic arthritis	F	38	Local steroid injection
3	Psoriatic arthritis	F	40	Prednisone
4	Seropositive RA	F	72	Methotrexate, tocilizumab
5	Polyarthrit unspec	M	24	Methotrexate
6	Oligoarthritis	M	49	Naproxen, local steroid injection
7	Psoriatic arthritis	F	66	Untreated
8	Juvenile arthritis	F	32	Golimumab
9	Pelvospondylitis	M	22	Diclofenac
10	Spondylitis	M	62	Adalimumab, methotrexate, prednisone
11	Spondyloarthritis	M	38	Methotrexate, prednisone, golimumab, ketoprofen
12	Psoriatic arthritis	F	69	Methotrexate, infliximab
13	Oligoarthritis	F	67	Methotrexate, ketoprofen
14	Seropositive RA	F	51	Prednisone, methotrexate
15	Juvenile arthritis	M	47	Methotrexate, prednisone, golimumab
16	Systemic lupus arthritis+juvenile arthritis	F	35	Prednisone, methotrexate
17	Sjögren's syndrome+polyarthritis	F	56	Abatacept, calcium carbonate/cholecalciferol
18	Seronegative, polyarthritis	F	26	Celecoxib, omeprazole

### Isolation of synovial fluid mononuclear cells or peripheral blood mononuclear cells

Freshly collected heparinized synovial fluid mononuclear cells (SFMCs) from patients and peripheral blood mononuclear cells (PBMCs) from both patients and controls were isolated by Ficoll-Paque density gradient centrifugation (GE Healthcare, Little Chalfont, United Kingdom) by standard procedures.

### Flow cytometry

Cells were stained with fluorochrome conjugated monoclonal antibodies: PerCP or APC (from Biolegend, San Diego, California, USA) or Brilliant Violet 421 (from BD Biosciences, Franklin Lakes, New Jersey, USA), antihuman CD3, clone UCHT1, together with PE conjugated anti-human TCR γδ, clone B1 (from Biolegend) or APC anti-human TCR γδ, clone B1 (BD Biosciences) and APC conjugated anti-human CD27, clone L128, and PE-cy7 conjugated anti-human CD45RA, clone L48 (both from BD Biosciences) for 30 min at 4°C. The cells were then stained with 7-aminoactinomycin D (7-AAD, BD Biosciences Pharmingen) for 10 min at 4°C for visualization of viable 7-AAD negative cells (7-AAD^-^) and analyzed on a BD FACS Sort using the CellQuest software (BD Biosciences) or Beckman Coulter Gallios. Further analysis was performed using the software FlowJo 9.4.10 (Tree Star Inc, Ashland, Oregon, USA). APC conjugated anti-human IFN-γ clone B27 (Biolegend) was used for intracellular staining.

### *In vitro* proliferation assay, stimulation with IPP and ethanol

To assess the proliferative response, SFMCs were stained with carboxyfluorescein succinimidyl ester, (CFSE, Invitrogen, Carlsbad, California, USA). Cells were incubated at 37°C for 15 minutes and washed twice in PBS (Sigma, St Louise, Missouri, USA)+0.5% fetal calf serum (Sigma). The proliferative response was calculated based on the fraction of γδ or αβ T cells with low CFSE intensity.

Cells (1×10^6^ cells/ml) were cultured for 4 and 7 days in 24 well plates (Sarstedt, Nümbrecht, Germany) in 1 ml RPMI-1640 medium (Sigma) supplemented with 5% heat-inactivated human serum (Sigma), 2 mM L-glutamine (Sigma), 100 U/ml penicillin (Sigma), 100 µg/ml streptomycin (Sigma) and 20 U/ml recombinant human interleukin 2 (Pepro Tech, Rocky Hill, New Jersey, USA) at 37°C in 5% CO_2_. The cells were stimulated at start of culturing with IPP (Sigma), range 0.00025–250 µM or risedronate from the University of Eastern Finland, range of 0.00001–1000 µM and ethanol in a range from 0.00015–15 vol/vol %. The number of viable cells, as assessed by Trypan blue (Sigma) staining, was counted under microscope after 4 and 7 days incubation. The percentage of γδ and αβ T cells was measured with flow cytometry at start and after 4 and 7 days of culture, and the absolute numbers of viable and proliferating CFSE^low^ γδ T cells were calculated. To obtain dose-response curves for survival, proliferation and expansion, cells were cultured in 96 well plates, 1×10^6^ cells/200 µL RPMI, and supplemented as indicated above.

### Intracellular staining

To measure intracellular cytokine accumulation, SFMCs were re-stimulated after 7 days culture for 6 hours with either 0.25 µM IPP, 0.015% ethanol, 0.25 µM IPP+0.015% ethanol or 20 ng/mL phorbol 12-myristate 13-acetate (PMA, Sigma)+0.1 µM ionomycin (Sigma), and the last 4.5 hours of the 6 hours with Golgi Stop (BD Biosciences) diluted 1∶1500. This was followed by standard procedures for fixation and permeabilization using BD Cytofix/Cytoperm (BD Biosciences) and staining.

### Data analysis - definitions and statistical analysis

The total number of γδ T cells was calculated as: % γδ T cells x total number of viable cells per well at the end of culturing. The expansion, or fold-increase, was calculated as the total number of γδ T cells at the end of culturing over the total number of γδ T cells at start. Proliferation and survival were calculated first as absolute number of CFSE^low^ and CFSE^high^ cells per well respectively at the end of culturing. Both fractional expansion, - proliferation and - survival are given by values normalized to unity.

Wilcoxon signed rank-test was used to analyze significance between groups. Values reported are means +/− SEM. Prism 6 (Graph Pad Software, Inc, La Jolla, USA) was used for statistical analysis.

For doubling times, the rate constant *k* was adjusted to fit the equation N cells_t_ = N cells_t = 0_×e*^k^* ^t^ to data, in which cell doubling time (*T*) is equal to ln 2/*k*.

Concentrations of IPP and ethanol were varied independently and in combination, and possible synergistic effects are presented in 3D surface response plots using Sigmaplot version 12.3.

Bell-shaped dose-response curves for IPP were analyzed by a simplified version of the homotropic two-state model, HOTSM, without spontaneous activity (parameter *L* = 0) [25].

## Results

### Low doses of ethanol enhance the proliferation and expansion of IPP stimulated γδ T cells

**Figure 1:**
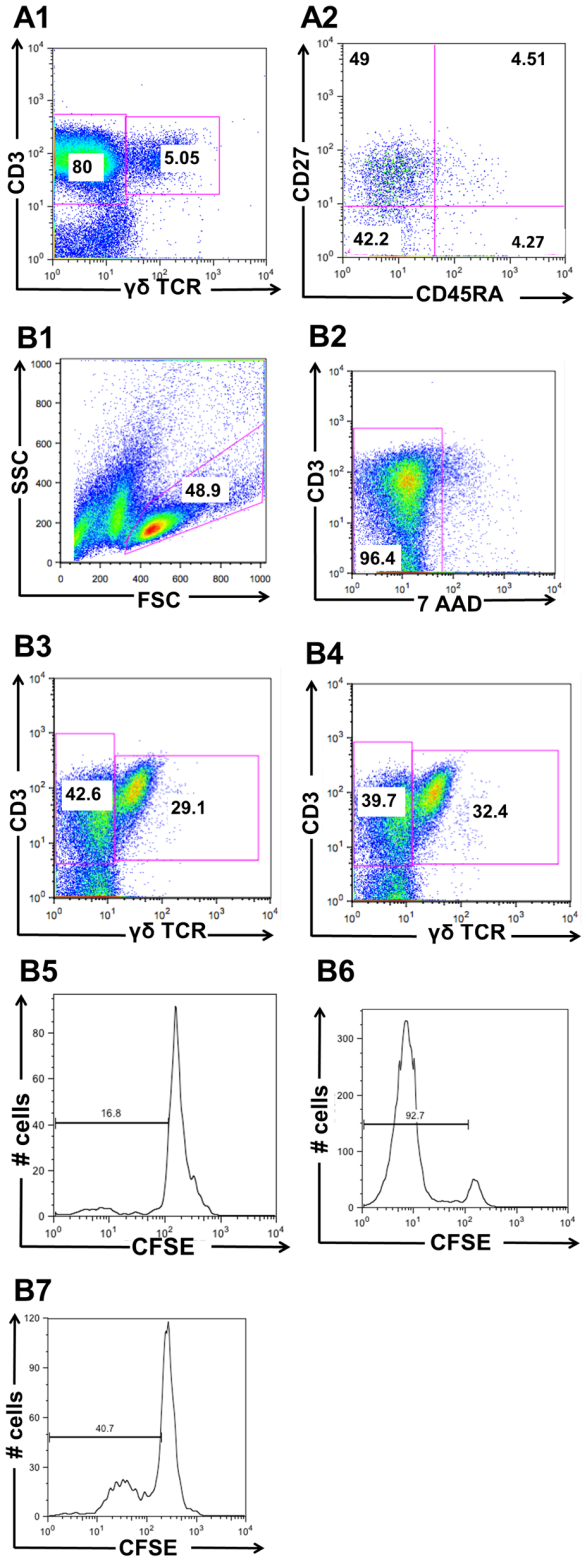
Phenotypic characterization and proliferative response of SFMCs. (**A**) SFMCs at start stained with antibodies to γδ TCR (A1) and γδ TCR gated cells stained with CD45RA and CD27 (A2). Representative FACS dot plots of two stainings. (**B**) Representative FACS dot plots of CFSE-labeled SFMCs in a single patient (pat no 10) after 7 days in culture. Gating of lymphocytes, FSC = Forward Scatter, SSC = Side scatter (B1). Gating of live 7-AAD^-^ lymphocytes (B2). Gating of γδ T cells stimulated with 0.25 µM IPP+IL2 (B3) and 0.25 µM IPP+0.015% ethanol+IL2 (B4). γδ T cells with different CFSE intensity bar show CFSE^low^ with no stimulation (B5) and with stimulation of 0.25 µM IPP+0.015% ethanol+IL2 after 7 days (B6) and after 4 days (B7).

The study was carried out to determine if low doses of ethanol could potentiate the effects of low doses of IPP in expansion of γδ T cells from inflamed joints. Thus, CFSE-stained SFMCs from patients with different arthritic diagnoses were stimulated *in vitro* with IPP and IPP in combination with ethanol, each in presence of IL2, for 4 and 7 days. The proportion of viable CFSE^low^ proliferating γδ T cells was measured by flow cytometry, Fig. 1, B5-B7. A concentration of ethanol at 0.015% was chosen as it represents the approximate blood concentration after one glass of wine or a shot of aquavit and is equivalent to the normal decrease in blood concentration per hour when ingested ethanol is metabolized [26].

**Figure 2:**
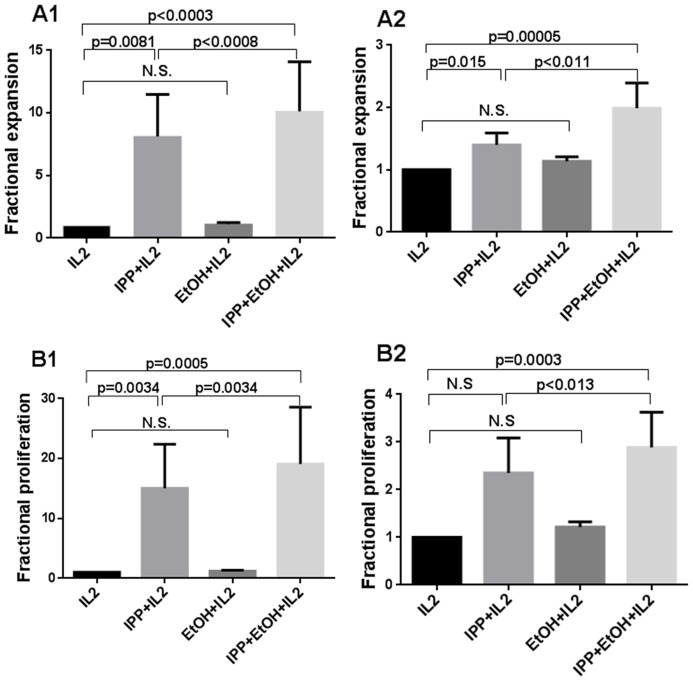
Expansion and proliferation of γδ T cells stimulated with IPP and ethanol for 7 days. SFMCs from patients and PBMCs from healthy controls were stimulated with 0.25 µM IPP, 0.015% ethanol or 0.25 µM IPP+0.015% ethanol, all in presence of IL2. Fractional A) expansion, showing fold-increase of total number of A1) SF γδ T cells from patients, n = 12 and A2) PB γδ T cells, n = 15 from healthy controls B) proliferation, showing total number of B1) CFSE^low^ SF γδ T cells from patients, n = 11 and B2) CFSE^low^ PB γδ T cells from healthy controls, n = 15, all normalized to IL2 = 1. The different growth conditions were compared using Wilcoxon signed rank test one-sided. Bars show means +/− SEM.

**Figure 3:**
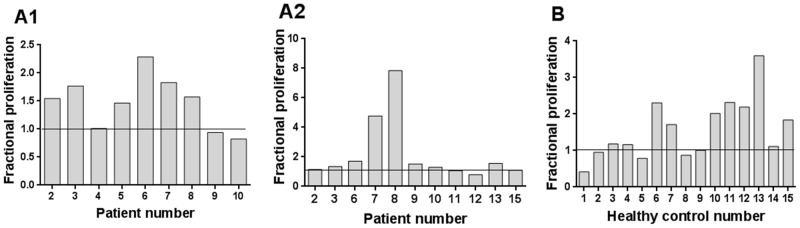
Relative proliferation of γδ T cells from different patients and healthy controls by adding 0.015% ethanol. Proliferation, showing total number of A) CFSE^low^ SF γδ T cells from patients incubated for A1) 4 days and A2) 7 days and B) CFSE^low^ PB γδ T cells from healthy controls incubated for 7 days, all normalized to 0.25 µM IPP = 1.

IPP, 0.25 µM, in combination with 0.015% ethanol gave a significantly higher fractional expansion, mean ratio 10.1+/−4.0 (p<0.0008, n = 12) and proliferation, mean ratio 19.1+/−9.5 (p = 0.0034, n = 11), compared with IPP alone after 7 days of SFMC culture, Fig. 2 A1 and B1. The effect of ethanol could be seen already after 4 days for expansion (p = 0.04) and for proliferation (p = 0.02) compared with IPP alone (n = 9), data not shown. In spite of a great variation in response to IPP between patients, Fig. 3 A1-A2, significant effects of ethanol were obtained in the rather small group of patients studied, Figs. 2 A1, B1 and 4. Considerably more patients would be needed to check for response differences between diagnostic subgroups. In addition, PBMC γδ T cells from healthy controls showed a higher fractional expansion, mean ratio 2.0+/−0.4 (p<0.011, n = 15) and proliferation, mean ratio 3.0+/−0.7 (p<0.013, n = 15) compared with IPP after 7 days, Fig. 2 A2 and B2. Similarly to the patient data, the effect of ethanol could be seen already after 4 days (data not shown), and a great variation was found in the response between subjects, Fig. 3 B.

### Effect of low doses of ethanol on cell-doubling time and survival of IPP stimulated γδ T cells

**Figure 4:**
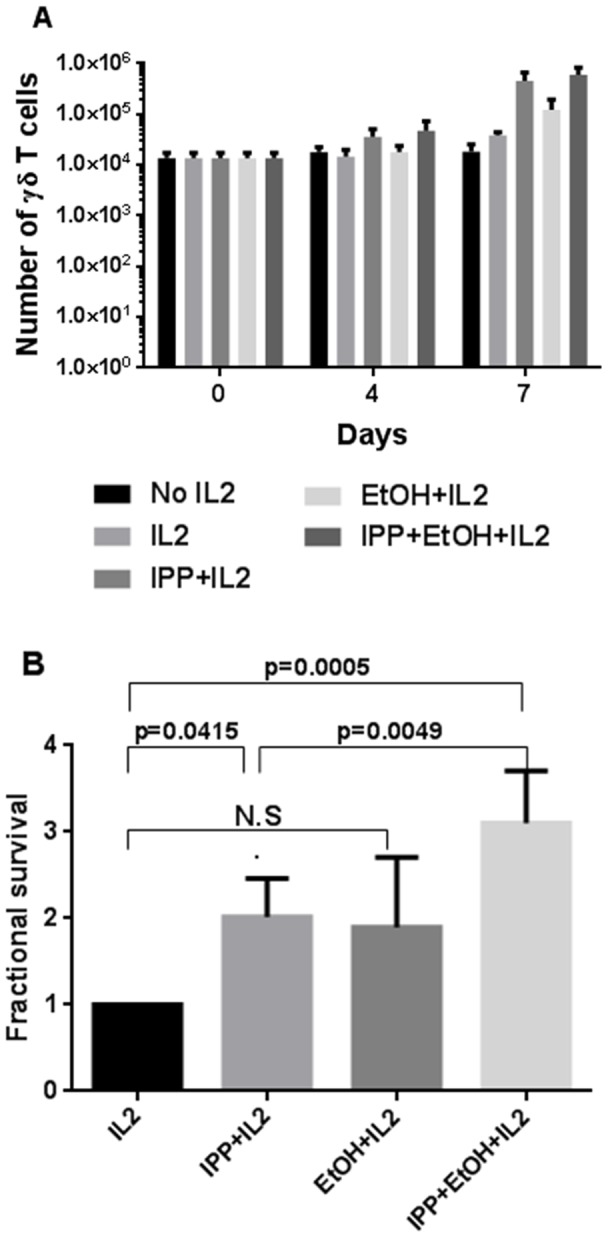
Growth data of total number of γδ T cells and survival of CFSE^high^ γδ T cells. A) SFMCs were untreated or treated with IL2 alone or together with 0.25 µM IPP, with 0.015% ethanol or with 0.25 µM IPP plus 0.015% ethanol for 7 days. Bars show means +/−SEM from five individual experiments. B) Relative response of adding 0.015% ethanol on the survival of CFSE^high^ γδ T cells stimulated for 7 days. SFMCs were stimulated with IL2 alone or in combination with 0.25 µM IPP, with 0.015% ethanol, or with 0.25 µM IPP+0.015% ethanol for 7 days. The number of surviving cells was calculated as viable γδ T cells CFSE^high^. Values are normalized to IL2 = 1. Wilcoxon signed rank test, one-sided, n = 11. Bars show means +/− SEM.

T cell proliferation may be analyzed by different factors such as initial cell death and cell doubling time [27]. To analyze the effects of low doses of ethanol on IPP stimulation of γδ T cells, we first investigated the cell population doubling time, equal to the first doubling time, using experimental growth curves as described in Materials and Methods. Doubling times are based on data for growth in figure 4 A. Adding 0.015% ethanol significantly reduces the cell doubling time from 52 to 34 hours, (p = 0.03, n = 5). Another factor we analyzed was the survival of cells. We found that by adding 0.015% ethanol, the number of surviving non-proliferating (7AAD^-^ CFSE^high^) γδ T cells after 7 days in culture was significantly higher compared to cells stimulated with IPP alone, normalized mean ratio 3.1+/−0.6 (p<0.005, n = 11), Fig. 4 B. Both the reduced doubling time and the augmented survival due to ethanol are part of the observed increase in IPP expansion induced by ethanol.

### Effect of ethanol on expansion of αβ T cells and on IFN-γ producing capacity

**Figure 5:**
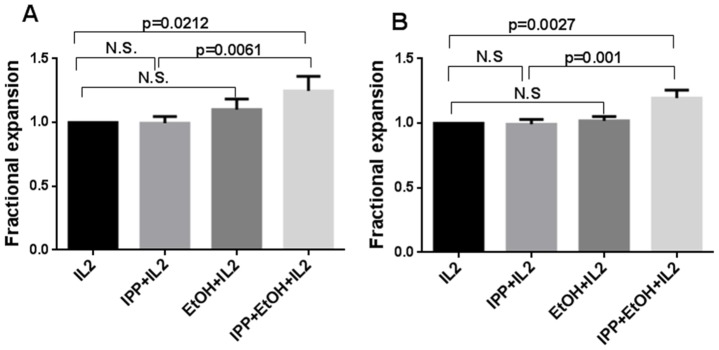
Effects of ethanol and IPP on expansion of αβ T cells. SFMCs from patients and PBMCs from healthy controls were stimulated with IL2 alone or in combination with 0.25 µM IPP or 0.25 µM IPP+0.015% ethanol for 7 days. Relative response of adding 0.015% ethanol on the expansion of A) SF αβ T cells from patients, n = 12 and B) PB αβ T cells from healthy controls, n = 15. Values are normalized to IL2 = 1. Bars show means +/− SEM.

We questioned if only γδ T cells were expanded and found that also αβ T cells from both patients and healthy controls were expanding when adding IPP and ethanol in combination. IPP alone had no effects on the expansion of αβ T cells above the effect of IL2, Fig. 5.

**Figure 6:**
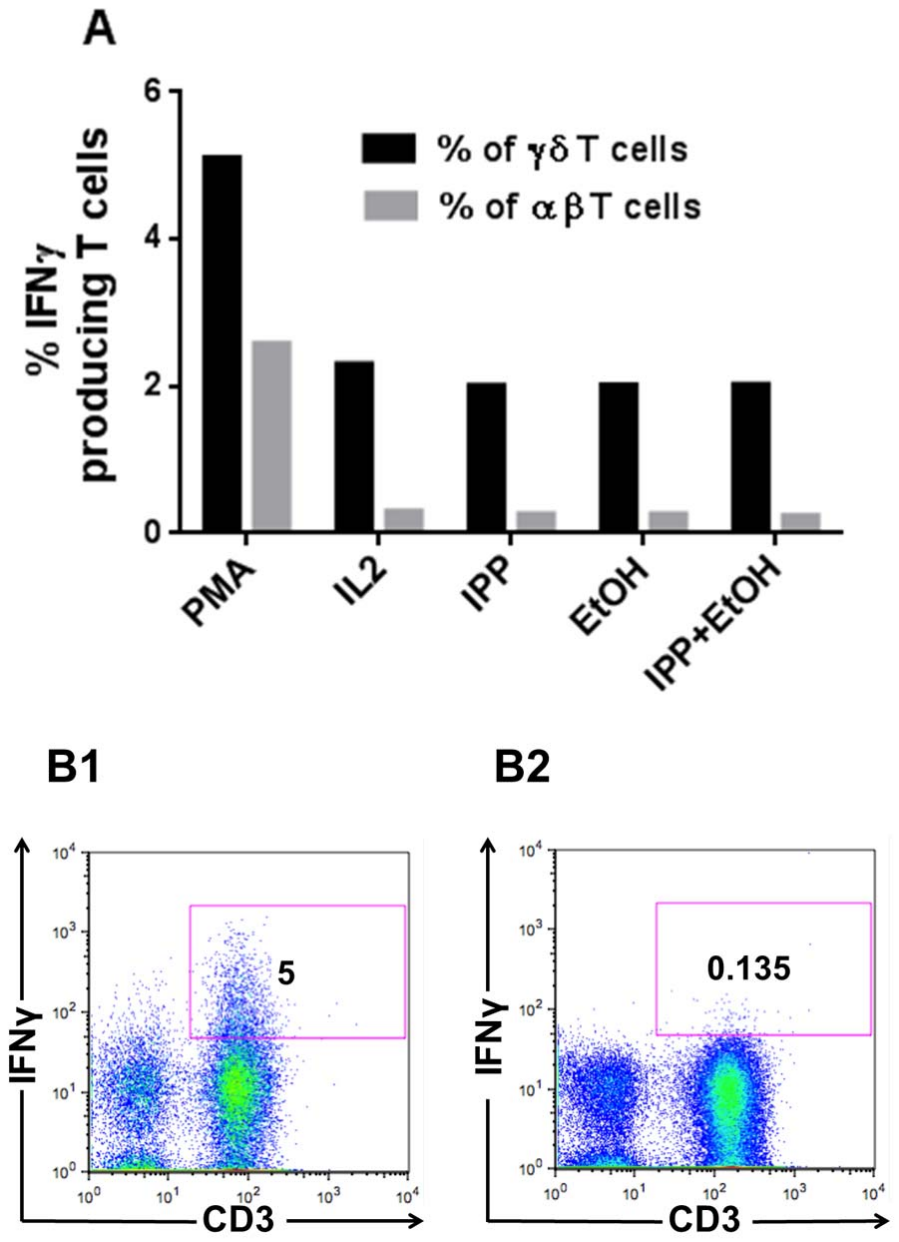
Effects of ethanol and IPP on cytokine production. SFMCs from patients were stimulated with IL2 alone or in combination with 0.25 µM IPP or 0.25 µM IPP+0.015% ethanol for 7 days. A) Effect on the number of IFN-γ producing SF T cells by adding 0.015% ethanol. Bars shows means, n = 5. Representative FACS dot plots of % IFN-γ producing T cells stimulated with PMA (B1) and 0.25 µM IPP+0.015% ethanol (B2).

We next asked what the effects of adding 0.015% ethanol had on the IFN-γ producing capacity. The percentage of IFN-γ producing SF T cells was not affected by 0.25 µM IPP or 0.015% ethanol compared with IL2 added alone, and the per cell production of IFN-γ was not elevated as measured by median fluorescence intensity, MFI, of IFN-γ producing SF T cells, Fig 6.

### Auto-inhibition by IPP

**Figure 7:**
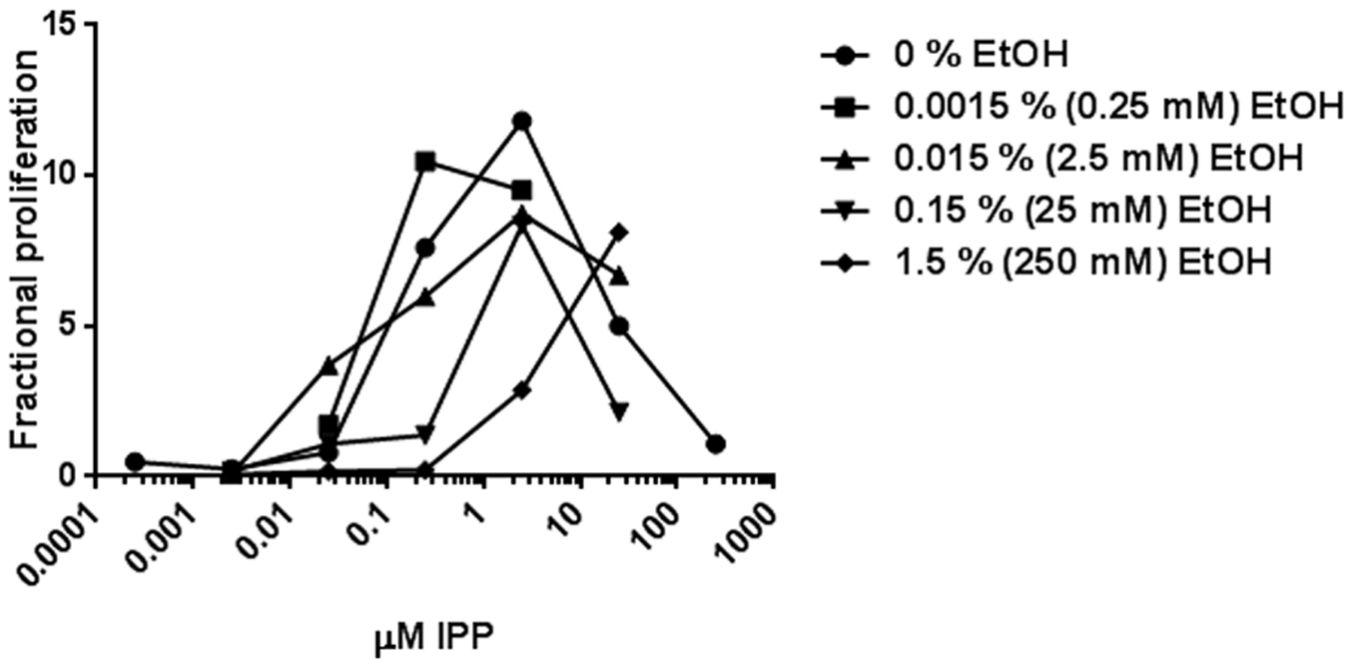
Effect of IPP in combination with ethanol on proliferation of γδ T cells. SFMC from patients with arthritis were stimulated for 7 days by varying concentrations of the agonist IPP and at different concentrations of the modulator ethanol together with IL2. The proliferations were determined for each combination. The data represents one single patient (no 12) and is one example of three performed experiments with similar results. Values are normalized to the absolute number of γδ T cells per culture well at start of experiment sat to unity.

Increasing IPP concentrations in absence of ethanol resulted in a bell-shaped dose-response curve for proliferation, Fig. 7. Such an auto-inhibition dose-response curve may indicate a receptive unit with two binding sites or two separate receptors for IPP of which an inhibitory site or receptor has the lowest association constant. The IPP auto-inhibitory relationship in figure 7 was analyzed with a homotropic two-state model, HOTSM [25]. Since the spontaneous activity in absence of IPP is almost zero and because data are limited, we used a simplified form of HOTSM with spontaneous activity equal to zero (*L* = 0). Equilibrium association constants for binding at a high affinity stimulatory site/receptor, *A*_s_, and at a low affinity inhibitory site/receptor, *A*_m_, were estimated by fitting of the HOTSM to data, yielding *A*_s_ = 5.37+/−0.96 and *A*_m_ = 0.91+/−3.32 (µM^−1^) combined with a binding cooperativity coefficient *c* = 0.105+/−1.48 and a maximum fractional proliferation factor at 18.47+/−0.80. These data for auto-inhibition by IPP is compared and discussed with similar observations in the literature, see Discussion.

### Synergistic and inhibitory effect of ethanol on proliferation of IPP stimulated γδ T cells

To further investigate the effects of ethanol on the proliferation of γδ T cells with IPP, we studied the auto-inhibition of IPP stimulated proliferation in the presence of ethanol. As shown in figure 7 there is a left-ward shift of the activated leg of IPP stimulated γδ T cells with low doses of ethanol. Since cotreatment with ethanol and IL2 did not stimulate γδ T cell expansion or proliferation more than IL2 alone as shown in Fig. 2, the ethanol enhancement of IPP stimulation resembles a synergistic rather than an additive effect of ethanol on IPP stimulation of γδ T cells. To document these potency effects of ethanol, we analyzed the shifts in dose-response curves of IPP-induced proliferation by ethanol with Michaelis-Menten kinetics. The potency effect, as an apparent decrease in the high-affinity EC_50_ values, is shown as an example in figure 7, where the leftward shift by adding 0.015% ethanol decreases the EC_50_ value fivefold from 0.19 µM+/−0.036 µM to 0.04 µM+/−0.019 µM, the same as a fivefold increase in affinity for IPP, or its potency. In case ethanol has activity in its own right and also lifts the max IPP effect, this is an allo-ago-synergy or additive effect. Alternatively, if ethanol does not stimulate in its own right but still lifts the max IPP effect, it is an allo-synergy or classical synergy effect [28]. However, there is a decrease in the max IPP effect, seen in figure 7 as a decrease in the maximum number of proliferating γδ T cells in proportion to γδ T cells at start from 12.83+/−0.61 to 8.15+/−0.75, indicating a lower efficacy with ethanol. Thus ethanol, cause both enhancement with a lower EC_50_ value (leftward-shift of the left leg) and efficacy inhibition (lower max IPP effect) [28].

### Response surface plots for concentration range of synergy

**Figure 8:**
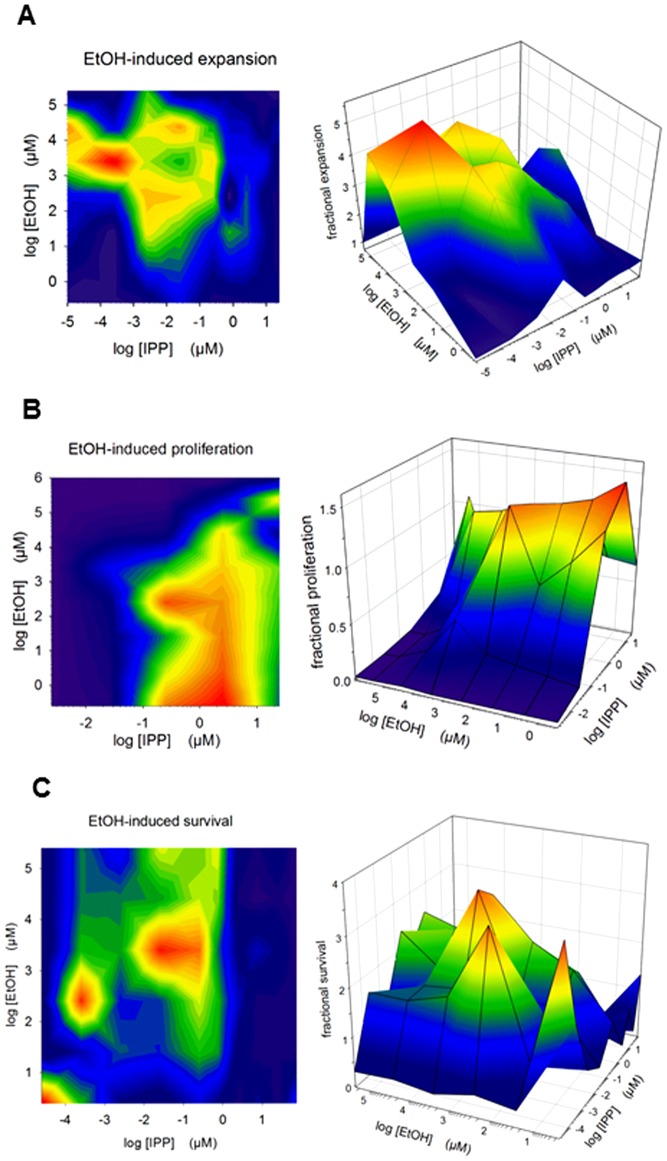
Effects of IPP in combination with ethanol on expansion, proliferation and survival of γδ T cells. SFMC from patients with arthritis were stimulated for 7 days with various concentrations of IPP and ethanol all in combination with IL2. The plots are representative of four independent experiments showing the fractional response normalized to 0.25 µM IPP = 1 for each combination, where the plots show A) expansion, data from patient no 15, B) proliferation, data from patient no 12 and C) survival, data from patient no 15.

To better demonstrate possible synergistic effects of ethanol at various combinations of drug concentrations, we performed experiments to create response surface plots. We first prepared dose-response curves to illustrate the effect of either IPP or ethanol alone on proliferation, survival and expansion. Concentrations of either IPP (agonist) or ethanol (modulator) were then varied, while keeping the other at a constant concentration. These results are shown as examples of expansion, proliferation and survival of SF γδ T cells in figure 8. These plots are more for illustrative purposes than for calculating parameters.

An example of expansion is shown in figure 8A, where the maximal effect (plateau) is lifted by ethanol at concentrations between 10^3^ and 10^4^ µM in combination with IPP concentrations of around 10^−3^ and 10^−4^ µM, demonstrating a clear synergistic effect of ethanol on IPP-expansion. An example of the effects of ethanol on proliferation can be seen in figure 8B, where a maximal peak effect by IPP around 10^−1^ µM IPP is lowered (in general) with ethanol concentrations above 10^3^ µM. Finally an example of survival is shown in figure 8C. IPP at low concentration in combination with ethanol can enhance the expansion, but as can be seen from the survival response surface plot, IPP and ethanol effects on survival have a rather complex interaction with several peaks indicating an intricate synergy. Presently we have no suggestion for possible mechanisms behind the observed synergy peaks on survival shown in figure 8C.

### Effect of ethanol on expansion and proliferation of risedronate stimulated γδ T cells

**Figure 9:**
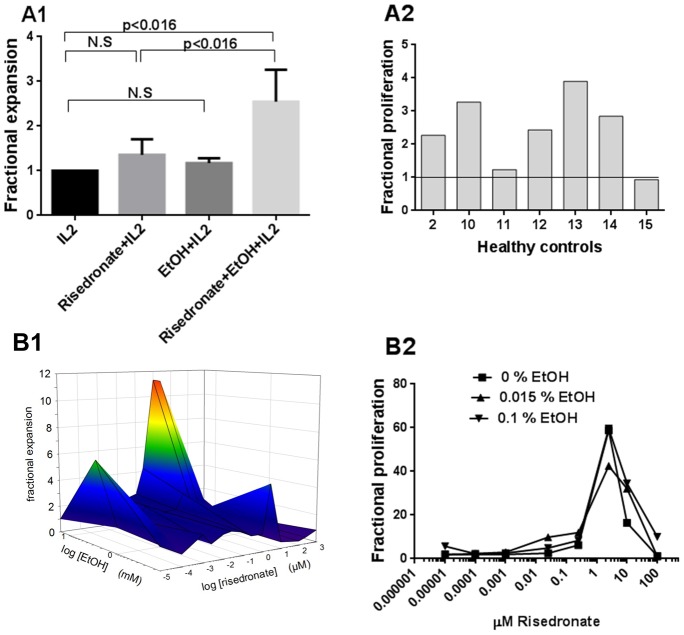
Expansion and proliferation of γδ T cells stimulated with risedronate and ethanol for 7 days. A) PBMCs from healthy controls were stimulated with 0.025 µM risedronate, 0.015% ethanol or 0.025 µM risedronate+0.015% ethanol, all in presence of IL2. A1) Fractional expansion, showing fold-increase of total number of PB γδ T cells, n = 6 from healthy controls. A2) Fractional proliferation, showing total number of CFSE^low^ PB γδ T cells from healthy controls, n = 7, all normalized to 0.025 µM risedronate = 1. The different growth conditions were compared using Wilcoxon signed rank test one-sided. Bars show means +/− SEM. B) PBMC from healthy controls were stimulated for 7 days with various concentrations of risedronate and ethanol all in combination with IL2. The plots are representative of three independent experiments showing the fractional response normalized to IL2 = 1 for each combination, where the plots show B1) expansion, data from healthy control no 2 and B2) proliferation, data from healthy control no 13.

It has been suggested that bisphosphonates stimulate γδ T cells indirectly by inhibiting farnesyl diphosphate synthase leading to the accumulation of endogenously produced IPP [17]. We therefore asked whether ethanol could enhance the expansion and proliferation of γδ T cells stimulated with bisphosphonate induced IPP. We found that ethanol has similar enhancing effect under these circumstances, Fig. 9. PBMC γδ T cells from healthy controls stimulated with 0.025 µM risedronate in combination with 0.015% ethanol showed a higher fractional expansion (p<0.016, n = 6, Fig. 9 A1) and proliferation (p<0.016, n = 7) compared with risedronate and IL2 alone. Ethanol and IL2 did not have any effects above IL2 alone. The effect of ethanol on risedronate stimulated γδ T cells could be seen already after 4 days (data not shown), and a great variation was found in the response between subjects, Fig. 9 A2. The enhancing effect of ethanol was further demonstrated in figure 9 B1–B2. The risedronate concentration 0.025 µM was chosen based on dose-response experiments, Fig. 9B and earlier studies [17], [29].

## Discussion

Our investigation shows that low to moderate doses of ethanol in presence of low concentrations of IPP can enhance the *in vitro* expansion of synovial fluid γδ T cells from patients with inflammatory arthritis. Several studies have documented the effect of ethanol on lymphocytes in blood from humans [24], [30]. It has been shown that ethanol can augment mitogen-induced T cell proliferation with phytomhemagglutinin (PHA) and concanavalin A (ConA) [24]. PHA and ConA have also been shown to be γδ T cell stimulatory reagents [31],[32] and PHA has been shown to give proliferative effect on T cells in synovial fluid [33]. It is therefore possible that ethanol may enhance or synergize other γδ T cell stimulatory reagents, beside IPP. To our knowledge, however, this is the first time results are obtained on ethanol stimulated proliferation of *synovial fluid* γδ T cells *in vitro* in humans at a blood alcohol concentration range equivalent to what is obtained with one or two glasses of wine. This knowledge may eventually lead to better diagnostics and treatment of arthritic diseases.

### Effects of IPP on expansion of SF γδ T cells

In this study SF γδ T cells showed proliferation capacity at 0.25 µM IPP, a response which is normally found for memory cells [34]. Indeed, γδ T cells from SF of patients with inflammatory arthritis are generally of a memory subtype [35]. There are essentially no cells in uninflamed healthy joint fluid [36], so control data from healthy patients are therefore not possible to present. However, our preliminary data indeed indicate that the ethanol enhancing effect is general, as we see similar effects on PBMC both from patients with arthritis (data not shown) as well as from healthy donors. Our results also show similar enhancing effects of ethanol on risedronate stimulated PBMCs from healthy donors. To our knowledge, there are currently no data available in the literature on the levels of IPP in plasma or in the inflamed joint *in vivo,* but farnesyl pyrophosphate has been measured in blood from healthy humans at a concentration of 0.017 µM [37], which is slightly below the concentration of IPP used in our study, 0.25 µM. SF αβ T cells from arthritic patients have shown a general hyporesponsiveness [38]. Meanwhile, other studies show proliferative response of SF γδ T cells in patients with arthritis stimulated with IPP [20], [23], synthetic alkyl phosphates [39] or with eubacteria using the MEP pathway [1-deoxy-D-xylulose 5-phosphate (DXP) pathway] such as *Mycobacterium tuberculosis* [33] and *Yersinia enterocolitica* [40], [41]. Further studies are needed to determine the IPP levels *in vivo* in the synovial fluid and the synovial lining in patients with arthritis.

### A possible homotropic allostery of IPP proliferation

Studying a range of IPP concentrations, we observed a bell-shaped dose-response of SF γδ T cell proliferation with a maximum at around 1 µM IPP, Fig. 7. We question if concentrations above 1 µM of IPP are toxic for SF γδ T cells or have a regulatory function, thereby balancing their proliferation. In case IPP at higher concentrations down-regulate proliferation it may be due to an inhibitory modulation through binding to a low affinity site. Bell-shaped dose-response curves are often observed in effectors as receptors, transporters and enzymes when an agonist itself binds to a modulator site and thereby affects its own function. Our observation of a bell-shaped proliferation induced by IPP is consistent with other studies, e.g., the study by Dieli et al [34] demonstrating bell-shaped IPP-induced proliferation of Vδ2 T cells in blood from healthy individuals. In another study, Tanaka and coworkers showed bell-shaped proliferation of SF γδ T cells with alkyl phosphates [39]. The lowered proliferation at higher IPP concentrations could be due to IPP binding to an inhibitory allosteric receptor site with low affinity, or due to a stimulatory allosteric site that activates cell death, possibly by death signals including Fas/FasL and others [42]. In Dieli's study [34] naïve γδ T cells show a bell-shaped dose-response as well, although it has been shown that naïve T cells do not express FasL [43]. Accordingly, we assume that there can be alternative explanations for the bell-shaped dose-response besides activation by Fas/FasL.

**Figure 10:**
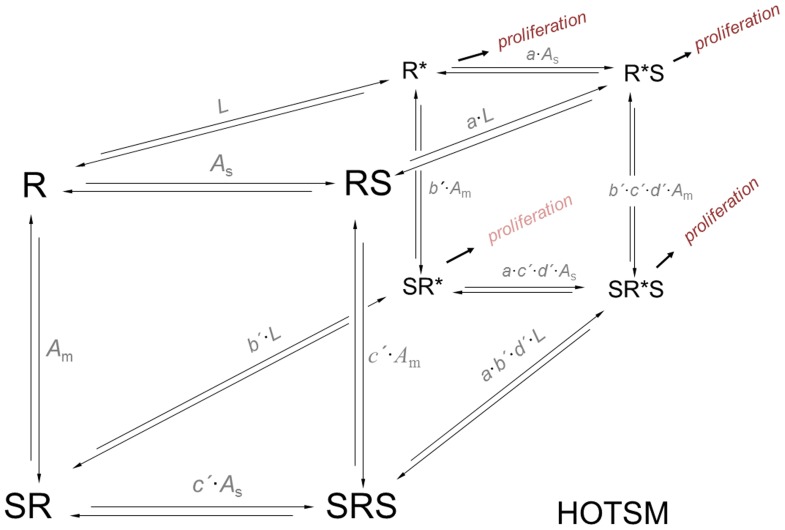
Homotrophic two-state model, HOTSM. An agonist or substrate, S, can bind to two sterically separate sites in a receptor R. An unbound receptor in such a system can be in two conformations, an inactive form, R, and active form, R*. The displayed HOTSM is a thermodynamically complete reaction scheme for such a system. Bell-shaped dose-response relations, as for the IPP effect on γδ T cell proliferation, may be analyzed according to this HOTSM scheme. S = agonist. R and R* are receptor in inactive and active conformations respectively. *L* = the isomerization constant between unbound R* and R, *L* = R*/R. *A*_s_ = the equilibrium association constant for S at the orthosteric site of R. *A*_m_ = is equilibrium association constant for S at the allosteric site of R. *a* = efficacy constant for RS 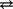 R*S when S is already bound to the orthosteric site. *b* = efficacy constant for SR 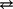 SR* when S is already bound to the allosteric site. *c* = co-operativity coefficient for binding of S at either site to an already liganded R. *d* = co-operativity coefficient for efficacy induced by one ligand on binding when another ligand is already bound to R*s or an efficacy coefficient when two ligands are already bound

Therefore, due to the lack of a clear explanation for the bi-phasic effect, we suggest that the observed bell-shaped IPP dose-response relation covers regulatory auto-inhibition, homotropic allostery, and may be analyzed by a homotropic two-state model, HOTSM. HOTSM is an auto-regulatory reaction scheme with spontaneous activity, Fig. 10 [25]. Table 2 lists fitted values for HOTSM parameters *A*_s_, *A*_m_ and *c* for the IPP bell-shaped response in our study. These values fall within the range of values for the same parameters extracted with the HOTSM from the study by Dieli et al [34]. We note that the values of *A*_s_, *A*_m_ and *c* for IPP from the bell-shaped profile in this study are within less than a factor of 10 of those for the memory CD45RA^-^CD27^+^ cells studied by Dieli et al. Much higher concentrations of IPP as well induced a bell-shaped proliferation of naïve Vδ2 T cells [34], probably due to lower affinity constants compared to memory cells. Thus, the same parameter values for naïve cells in their study are off by factors of more than 500, Table 2.

**Table 2:** Best fit values for parameters of the homotropic two-state model, HOTSM, based on IPP-proliferation of γδ T cells.

Parameter	Dieli 1 (memory)	Present study	Dieli 2 (naïve)
*A*_s_ (µM^−1^)	52.2	5.37	0.0104
*A*_m_ (µM^−1^)	0.321	0.912	0.000309
*c* (factor)	0.373	0.105	0.350
Proliferation	52.8 (cpm·10^−3^)	18.5 (fractional)	29.4 (cpm·10^−3^)

Values for equilibrium association constants *A*_s_ and *A*_m_ and the cooperative binding constant *c* were extracted by fitting the HOTSM relation [25] to bell-shaped dose-response curves of IPP activation-inhibition of γδ T cell proliferation from this study and data in the literature [34], Fig. 4A. As our experiments indicated almost no response in the absence of ligands IPP and EtOH, parameter *L* was kept at zero symbolizing no spontaneous activity. Values for our proliferative response of T cells - most likely as memory CD45RA^-^CD27^+^ cells - to IPP in a concentration range from 3×10^−3^ to 3×10^2^ µM are within a factor of 10 to the data set for memory cells in Dieli et al's 2003 study, columns 2 and 1.

### A possible mechanism for synergistic effects of ethanol and IPP

Ethanol may act not only through the disordering of lipids in cell membranes [44], but also more specifically on enzymes and ion channels [45]. Ethanol has low binding affinity to proteins, thus normally concentrations 15 to 150 mM are required for detectable binding, although alcohol binding to alcohol-dehydrogenase is in a low millimolar concentration range [45].

With low doses of IPP present, we show an increased proliferation of SF γδ T cells by adding a moderate dose of 0.015% (2.5 mM) ethanol, Fig. 2B1 and 3A. This concentration of alcohol lies within the blood alcohol concentration range reached when alcohol is consumed socially, while it is more than three orders above the normal 15 µM of ethanol in blood [46]. Further, we show that SF γδ T cells expand more, Fig. 4A, that non-proliferating SF γδ T cells survive better, Fig. 4B, and that SF αβ T cells increase in numbers when a low dose of ethanol is added together with IPP, Fig. 5A.

Figure 7 shows the effects of combining ethanol with the IPP-induced bell-shaped response on proliferation of SF γδ T cells. In the presence of moderate doses of ethanol, the width of the IPP bell-shaped response is broadened both left and right. At the bell's left leg with low doses of IPP present there is an enhanced proliferation at moderate doses of ethanol on the expansion of SF γδ T cells. This indicates an ethanol-induced increase in affinity for IPP. At the bell's right leg with high doses of IPP, there is a shift to the right in the presence of moderate doses of ethanol. This points to a decrease in the IPP affinity at an inhibitory site for IPP, elicited as a mixed inhibition by ethanol. In the HOTSM for IPP, these effects of alcohol may be interpreted as an increase in parameter *A*_s_ (1/EC_50_) and a decrease in parameter *A*_m_ (1/IC_50_) for IPP when adding ethanol. Further, the peak of the IPP-induced bell-shaped response is lowered by moderated doses of ethanol, Fig. 7. This may be interpreted either as an effect on the cooperative binding coefficient *c* for IPP induced by ethanol at both IPP binding sites or as an ethanol-induced proportional down-regulation of both types of IPP receptors due to the symmetry of reduction. Thus, if our model for the combined effects of IPP and ethanol is valid, ethanol binding at a single site elicits synergistic conformational changes in both a stimulatory and an inhibitory site for IPP-regulated proliferation.

Meanwhile, we cannot exclude that the shift to the left by ethanol of the bell-shaped curve in figure 7 is due to a bitopic ligand formed by IPP and ethanol or their metabolites such as acetaldehyde. Our results show that ethanol has an enhancing effect on IPP stimulated γδ T cell expansion, also when endogenous IPP is generated by pharmacological means, using risedronate. Earlier studies have shown that IPP generated from bisphosphonates may react with metabolites forming new compounds [47]. Future studies may clarify if this may be an explanation for our results. Alternatively, the observed left-ward shift may indicate binding sites at low concentrations of both compounds, where a putative binding site for ethanol is either upstream or downstream of the binding sites for IPP proliferation.

When increasing the concentration of ethanol above 25 mM, the IPP bell-shaped response in figure 7 is lowered from its maximum. We would have expected an increase in proliferation of IPP stimulated γδ T cells at these concentrations since earlier studies have shown an enhancing effect on mitogen-activated T cells at a concentration of 150 mM ethanol [24]. Our findings at the higher ligand concentrations indicate mixed competition between IPP and ethanol [28]. Other studies have shown that ethanol by stimulating cyclic adenosine monophosphate (cAMP) can activate protein kinase A (PKA) which would block γδ T cell proliferation via the mitogen-activated protein kinase (MAPK) pathway. Meanwhile, ethanol up to 200 mM may have an effect on cAMP without activating PKA [24], [48]. It is possible that the effects of ethanol on cAMP in the presence of IPP can give signals via MAPK by a different route such as via exchange proteins activated by cAMP (EPAC), or protein kinase C (PKC) and escape the blockade from PKA [49], [50], thereby also overcoming the inhibitory effects of prostaglandin E2 (PGE_2_) on γδ T cell proliferation and cytotoxicity described in other studies [51], [52].

### Effects of low concentrations of ethanol and IPP on proliferation of different T cell populations

Ethanol may have different effects on different T cell populations. Other studies have shown that ethanol at 150 mM *in vitro* can augment mitogen-induced T cell proliferation in human blood stimulated with mitogens, ConA or PHA [24], but a lower concentration of 25 mM of ethanol could at the same time inhibit proliferation of activated antigen-specific T cells [24]. Because γδ T cells are not MHC dependent, an enhancing effect of ethanol on expansion of γδ T cells together with IPP could be expected. In this study we also found an increased proliferation of αβ T cells when low to moderate levels of ethanol together with low concentration of IPP was added. Transient increases in αβ T cells have also been found in studies of macaques when stimulated with HMBPP [53]. It is conceivable that if γδ T cells secrete IL2, an increased number of IL2 secreting SF γδ T cells could also stimulate an expansion of SF αβ T cells.

### Effects of ethanol on cytokine production

Central memory T_CM_ cells (CD45RA^-^CD27^+^) can be further differentiated into different effector subgroups such as effector memory T_EMh_ cells (CD45RA^-^CD27^-^) which are perforin^low^ IFN-γ^high^, resembling the Th1 cells, or the more cytotoxic T_EMRA_ cells (CD45RA^+^CD27^-^) which are IFN-γ^low^ and perforin^high^ [54]. In one study, IPP stimulation of SF cells from juvenile arthritis gave an increased proportion of IFN-γ cells [23]. Another study has shown that stimulation with IPP only of memory PB γδ T CD45RA^-^CD27^+^ cells in arthritis expands T_EM_ CD45RA^-^CD27^-^ after 7–14 days in culture [35]. In contrast, in our study we found that SF γδ and αβ T cells did not respond with increased IFN-γ secretion when stimulated with the lower concentrations of IPP with or without ethanol. Our preliminary results show instead that adding ethanol gives the more cytotoxic perforin producing T_EMRA_ cells (preliminary results, data not shown). Effector cytokines such as perforin produced at the peak expansion coinciding with the increase in αβ T has been found also in the study with macaques stimulated with HMBPP [53].

The benefit of alcohol to treat rheumatoid arthritis has been known for a long time. Aquavit as a remedy for alleviation of arthritic pain was described already in a booklet from 1843 [55]. “Drink a spoonful each morning for several weeks and scrub it with help of a brush to the painful parts each morning and evening”. What the inventor of the aquavit treatment, William Lee, did not know, was that his prescription may have an effect on the activation of γδ T cells. Indeed the prescribed amount of ethanol is likely to be within the range of a stimulating concentration on Vδ2 T cells. Thus, our findings provide a possible mechanistic explanation for the historical observation of William Lee.

In conclusion, we have demonstrated that an ethanol concentration equivalent to one glass of wine can enhance expansion of IPP stimulated γδ T cells. If these expanded γδ T cells can target FLS this could be one explanation of the protective role of ethanol in arthritis and possibly in other diseases as well.
